# Enhancing glaucoma care with smart contact lenses: An overview of recent developments

**DOI:** 10.1007/s10544-025-00740-7

**Published:** 2025-04-21

**Authors:** Ali Fardoost, Koosha Karimi, Jaydeep Singh, Heneil Patel, Mehdi Javanmard

**Affiliations:** Department of Electrical Engineering, Rutgers University, 08854 Piscataway, NJ USA

**Keywords:** Smart contact lenses, Glaucoma, Intraocular pressure (IOP) monitoring, Biosensors, Biocompatible materials, Drug delivery systems, Continuous monitoring, Ocular health

## Abstract

Glaucoma is a leading cause of irreversible blindness worldwide, affecting millions of individuals due to its progressive damage to the optic nerve, often caused by elevated intraocular pressure (IOP). Conventional methods of IOP monitoring, such as tonometry, provide sporadic and often inaccurate readings due to fluctuations throughout the day, leaving significant gaps in diagnosis and treatment. This review explores the transformative potential of smart contact lenses equipped with continuous IOP monitoring and therapeutic capabilities. These lenses integrate advanced materials such as graphene, nanogels, and magnetic oxide nanosheets alongside sophisticated biosensing and wireless communication systems. By offering continuous, real-time data, these lenses can detect subtle IOP fluctuations and provide immediate feedback to patients and clinicians. Moreover, drug-eluting capabilities embedded in these lenses present a groundbreaking approach to glaucoma therapy by improving medication adherence and providing controlled drug release directly to the eye. Beyond IOP management, these innovations also pave the way for monitoring biochemical markers and other ocular diseases. Challenges such as biocompatibility, long-term wearability, and affordability remain, but the integration of cutting-edge technologies in smart contact lenses signifies a paradigm shift in glaucoma care. These developments hold immense promise for advancing personalized medicine, improving patient outcomes, and mitigating the global burden of blindness.

## Introduction

Glaucoma, often termed the "silent killer of vision," is a leading cause of irreversible blindness, affecting nearly 3 million individuals in the United States and 80 million people globally, with a survival rate of about 90% (Quigley and Broman 2006). It is characterized by progressive damage to the optic nerve, primarily caused by elevated intraocular pressure (IOP) (Wang et al. 2023). Elevated IOP results from impaired drainage of aqueous humor, a clear fluid responsible for maintaining eye pressure. When the trabecular meshwork-the drainage system located at the junction of the iris and sclera-fails, fluid accumulation occurs, leading to optic nerve damage and eventual vision loss (Weinreb and Khaw 2004; Wang et al. 2023). Despite advancements in understanding glaucoma, the biological mechanisms underlying its progression remain largely undefined (Nickells et al. 2012).

IOP elevation is the most significant risk factor for glaucoma progression (Liu et al. 2009). However, early stages of the disease are asymptomatic, earning glaucoma the moniker “silent thief of sight.” This often results in patients experiencing substantial vision loss before symptoms become apparent. Regular IOP monitoring is crucial for delaying or preventing optic nerve damage (Sit et al. 2011). Common clinical tools for IOP measurement include the Goldmann applanation tonometer (GAT), non-contact tonometers (NCTs), and rebound tonometers (Konstas et al. 2006). These instruments guide treatment decisions, which may involve topical medications such as prostaglandin analogs, beta-blockers, or carbonic anhydrase inhibitors. Severe cases may require surgical interventions like trabeculectomy or the implantation of drainage devices, such as Ahmed or Molteno valves, to control fluid outflow and reduce IOP (Gedde et al. 2012).

Despite the utility of these conventional methods, periodic in-office measurements are insufficient to capture IOP’s significant diurnal variations, especially nocturnal spikes when pressure is often highest (Liu et al. 2009; Wilensky et al. 1993). Such fluctuations can exacerbate optic nerve damage and remain undetected, underscoring the need for continuous, real-time IOP monitoring (Asrani et al. 2000).

Continuous monitoring technologies have been developed to address these limitations, with smart contact lenses emerging as a groundbreaking innovation. These lenses, such as the Sensimed Triggerfish®, integrate biosensors to measure IOP continuously over 24 hours (Zhang et al. 2022). Using a strain gauge embedded in the lens, the device detects circumferential corneal changes caused by IOP fluctuations. Such technology provides clinicians with valuable nocturnal IOP data, enabling earlier detection of pressure spikes and timely treatment adjustments (Wu et al. 2024).

Beyond monitoring, smart contact lenses are being developed to include drug delivery systems. These lenses allow controlled release of IOP-lowering medications, such as timolol or latanoprost, directly to the eye, enhancing drug efficacy while improving patient compliance (Ciolino et al. 2014; Liu et al. 2022). This advancement addresses a critical challenge in glaucoma management, where adherence to topical medication regimens remains suboptimal (Ciolino et al. 2016; Powell et al. 2020).

Additionally, these technologies hold potential for managing secondary conditions associated with elevated IOP. For example, post-cataract surgery IOP reduction is particularly relevant for patients with narrow-angle glaucoma (Manoharan et al. 2018; Young et al. 2019). Similarly, continuous IOP monitoring could benefit patients with anterior uveitis or retinal vein occlusion, conditions where elevated IOP exacerbates disease progression (Wong and Scott 2010).

The future of glaucoma care lies in the integration of multifunctional smart contact lenses that combine continuous IOP monitoring, drug delivery, and biomarker detection. These devices represent a transformative shift toward personalized glaucoma management, offering tailored, data-driven treatment solutions (Wu et al. 2024; Kim et al. 2022). As research advances in optimizing material properties, functionality, and affordability, these lenses are poised to significantly reduce the global burden of vision loss associated with glaucoma.

## Industry standard tonometry

The history of tonometry dates back to 1862, with early devices based on the Imbert-Fick principle. Adolf Fick developed a spring-loaded tonometer for accurate intraocular pressure (IOP) readings in animal models, but it was not tested on humans (Mark 2011). In 1892, the Maklakoff tonometer became widely used, though it suffered from large applanation areas and inaccuracies (Kniestedt et al. 2008). The Schiotz tonometer, introduced in 1905, improved IOP measurements through indentation (Albert et al. 2020). However, continuous IOP monitoring remained elusive until the Goldmann Applanation Tonometer (GAT) revolutionized the field in the 1950s (Moses 1958).

The Goldmann Applanation Tonometry (GAT) has long been regarded as the gold standard for intraocular pressure (IOP) monitoring in glaucoma management due to its superior accuracy compared to other tonometric devices (Brusini et al. 2021). The GAT operates on the basis of the Imbert-Fick principle (Stamper 2011), expressed mathematically as:

1 $$\begin{aligned} P = \frac{F}{A} \end{aligned}$$

**Fig. 1:**
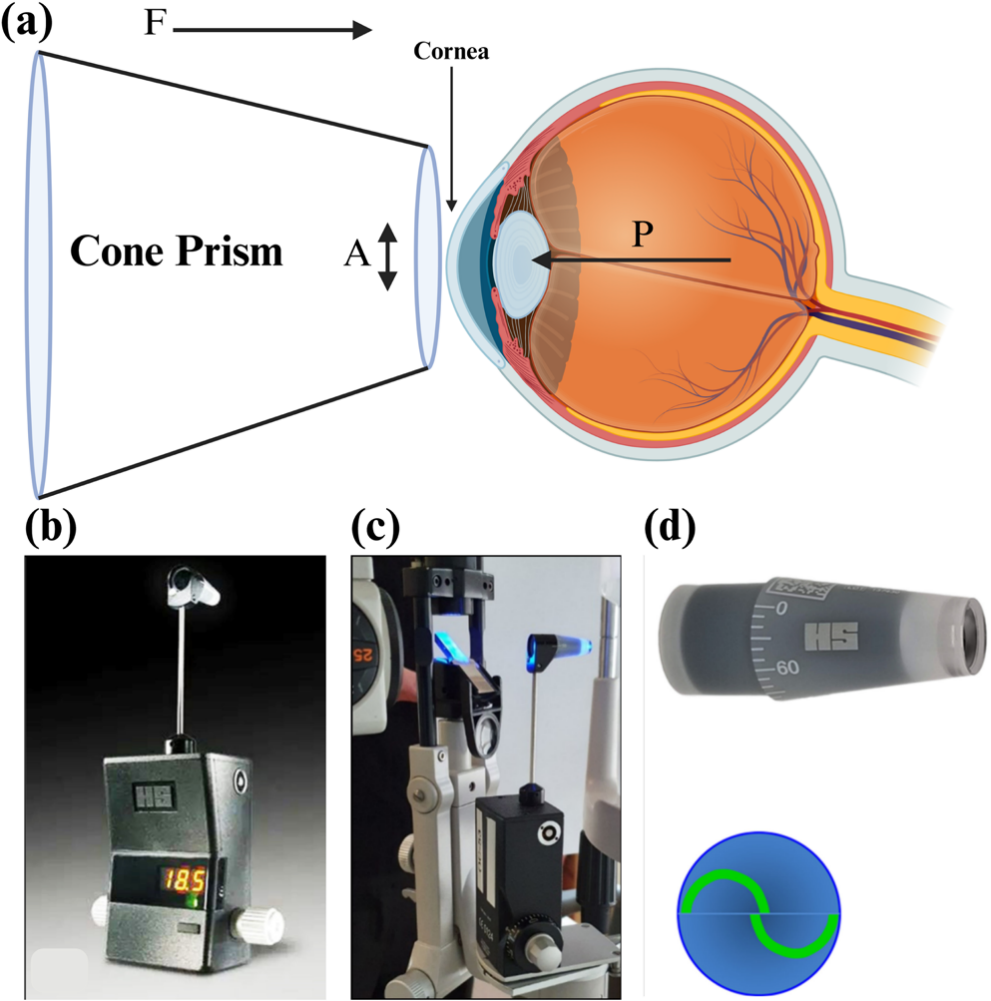
(a) Illustration of the GAT method. In this method, a cone-shaped prism exerts a force F onto the cornea. This applied force results in the deformation of the corneal surface, causing it to flatten to a surface area A. Consequently, the intraocular pressure (IOP) is determined by the relationship P=F/A. (b) digital Goldmann tonometer. (c) Goldmann applanation tonometer positioned on the slit lamp. (d) Cone prism on the top and two applanation rings being calibrated to become an ’S’ shape on the bottom (Brusini et al. 2021)

The GAT, based on the Imbert-Fick principle, remains the gold standard for IOP monitoring due to its accuracy (Brusini et al. 2021). It measures IOP by applying a probe to the cornea and calculating pressure based on force and area (Garcia-Feijoo et al. 2015), as shown in Fig. 1. Despite its precision, the GAT requires an anesthetic and manual calibration, as depicted in Fig. 1(d), and provides readings in 0.2 mmHg increments (Damji et al. 2003). The digital Goldmann Applanation Tonometer (dGAT) enhances precision with digital readings at 0.1 mmHg increments (Sakaue et al. 2014). A comparative study showed a strong correlation between GAT and dGAT readings, confirming dGAT’s accuracy for glaucoma management (Sakaue et al. 2014).

**Fig. 2:**
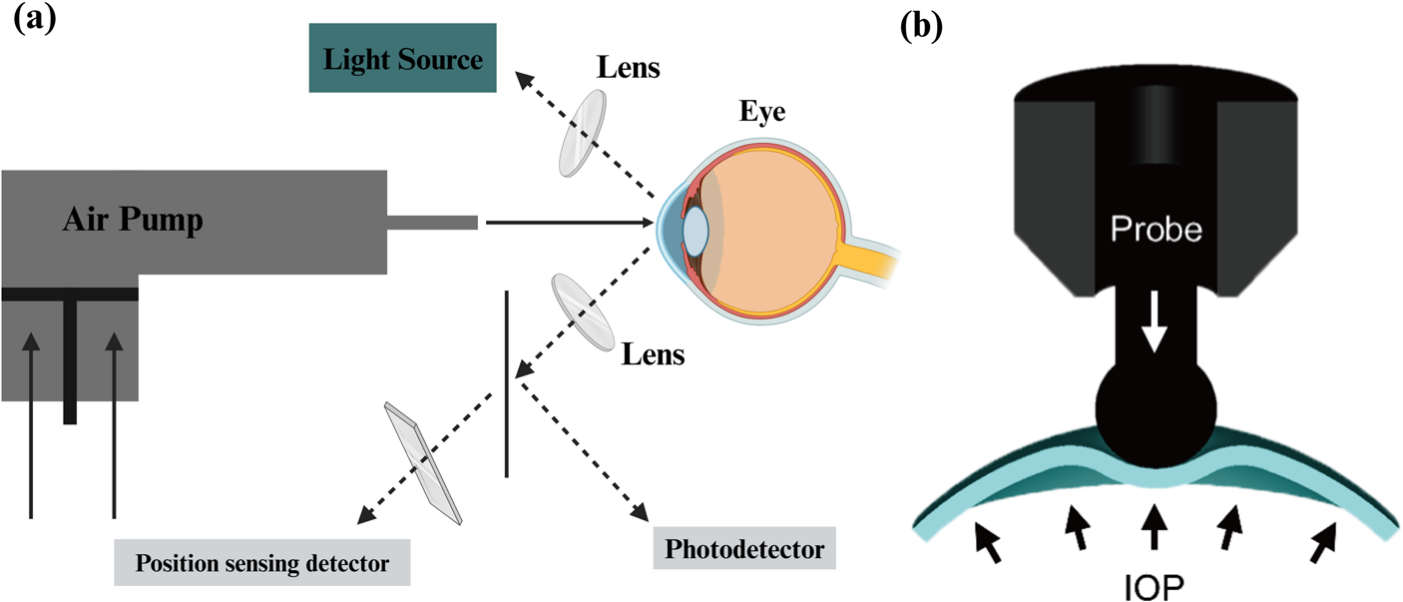
(a) The principle of the noncontact tonometry operation. An air pump puffs the air straight to the cornea surface, and the reflection of this air is captured to measure the IOP. (b) The schematic of the rebound tonometer includes a probe going toward the cornea surface, and the deceleration of the probe on its way toward the cornea after touching the surface measures the IOP (Kim et al. 2017)

While GAT is reliable, it has limitations such as lack of portability and the need for operator assistance (Grewal et al. 2016). New alternatives include electronic applanation tonometers, noncontact tonometers (NCTs), and rebound tonometers, which offer solutions for continuous IOP monitoring (Kaufmann et al. 2004; Lin et al. 2023). NCTs use pressurized air to flatten the cornea, while rebound tonometers measure the deceleration of a probe to estimate IOP (Figure 2) (Kim et al. 2017).

Though promising, NCTs and rebound tonometers face accuracy concerns, particularly in cases of elevated IOP (Chen et al. 2019). Studies have shown significant variability in NCT readings compared to GAT in patients with high tear meniscus heights and glaucoma (Seol et al. 2019). Rebound tonometers, while portable and practical in settings without anesthetic, can also show inconsistencies in high IOP cases.

Despite these advances, continuous IOP monitoring remains a challenge, leading to the development of smart contact lens tonometers (Zhang et al. 2022). These devices offer real-time IOP tracking and molecular sensing, representing the next frontier in glaucoma care (Wu et al. 2024).

## Materials utilized in the production of smart contact lenses

The design of contact lenses for glaucoma management and broader ocular health hinges on biocompatibility, which ensures comfort, reduces irritation and allows continuous monitoring (Yadav et al. 2024; Kazanskiy et al. 2023). Essential properties for biocompatibility include softness to prevent inflammation, transparency for vision preservation, oxygen permeability, and wettability to avoid dryness (Ferraz 2022; Chaudhari et al. 2021; Karayilan et al. 2021). Advances in materials have driven the development of smart lenses for glaucoma care, particularly for monitoring intraocular pressure (IOP) (Han et al. 2024). The following sections review key studies on biocompatibility and material innovations guiding the design of these lenses.

### Gold and silver nanoparticles

Salih et al. (2022) introduced a method to embed gold and silver nanoparticles into commercial contact lenses using a "breathing-in/breathing-out" (BI-BO) technique. Unlike traditional methods, their approach loads nanoparticles post-manufacture, preserving the lenses’ material properties such as wettability and biocompatibility.

The BI-BO method involves placing hydrated lenses in an aprotic solvent to remove water, followed by rehydration in a nanoparticle solution, allowing controlled nanoparticle loading. This tunable process affects optical properties and is scalable.

Salih et al. demonstrated that silver nanocomposite lenses filter blue light (400-450 nm) effectively, comparable to commercial blue-light blocking wearables, while gold nanocomposites filtered red-green overlap wavelengths (522 nm), aiding in colorblindness correction (Salih et al. 2021). No nanoparticle leaching was observed over a month, ensuring stability and safety.

This study presents a practical method for enhancing contact lenses, expanding their potential in healthcare and nanocomposite lens applications.

### Inorganic $\gamma \text {-}\mathrm {Fe_2O_3}@\textrm{NiO}$ magnetic oxide nanosheets

**Fig. 3:**
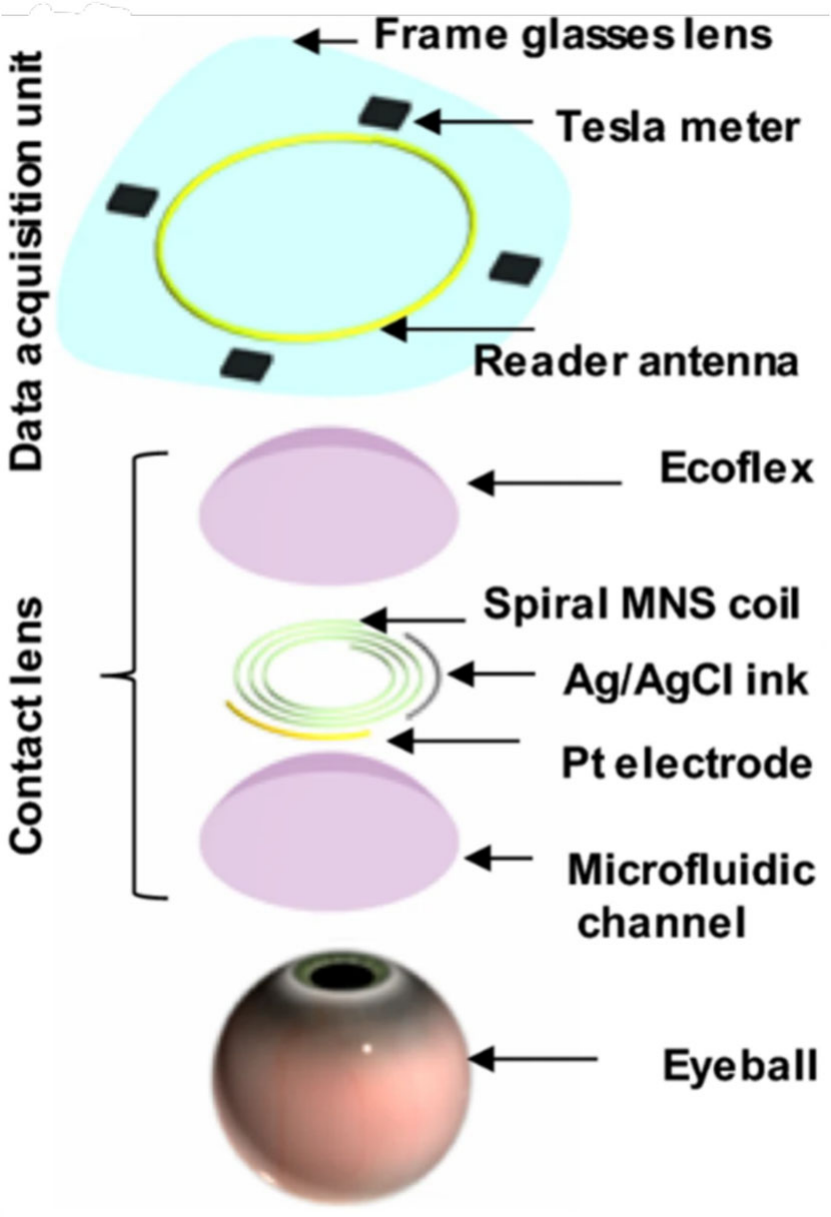
Diagram illustrating the primary components of the MCL, including a microfluidic channel, Pt electrode, spiral MNS coil, and Ecoflex. And the data acquisition unit (Xie et al. 2022)

Xie et al. (2022) developed an innovative multifunctional contact lens (MCL) using inorganic $\gamma \text {-}\mathrm {Fe_2O_3}@\textrm{NiO}$ magnetic oxide nanosheets (MNS) for real-time, non-invasive monitoring of chronic eye conditions. This MCL integrates biochemical and biophysical sensors to monitor tear glucose, intraocular pressure (IOP), and eye movement simultaneously, as shown in Fig. 3.

The use of $\gamma \text {-}\mathrm {Fe_2O_3}@\textrm{NiO}$ nanosheets leverages their magnetic and electrochemical properties, allowing for a multi-functional sensing material that retains flexibility and biocompatibility. Their hydrothermal synthesis produced highly conductive, porous nanosheets, achieving sensitive glucose detection with a low limit of $0.43 \ \mu \text {mol}$. This enzyme-free sensor demonstrated stability and selectivity, addressing tear glucose monitoring challenges.

For eye movement tracking, the lens’s magnetic properties achieved 95.27% accuracy, outperforming conventional sensors in simplicity and portability. The MCL’s spiral structure and wireless IOP sensor offer a non-invasive method for real-time IOP monitoring with high sensitivity (0.17 MHz/mmHg) over a broad range.

This work significantly advances smart contact lenses for personalized eye health monitoring, offering continuous multi-functional tracking in a flexible, biocompatible device. It presents a key development in wearable diagnostic technologies for managing conditions like diabetes, glaucoma, and eye vergence disorders.

### Graphene

Graphene is emerging as a revolutionary material for contact lenses, particularly in eye care and glaucoma management, as explored by Zhang, Y., Chen, Y., Man, T., and others (Zhang et al. 2019). Studies have utilized graphene to create woven fabrics (GWFs) with excellent strain resistance, flexibility, and transparency-key properties that enhance biocompatibility in contact lenses (Pang et al. 2019). The process involves growing graphene on a copper mesh via chemical vapor deposition (CVD), followed by chemical etching to remove copper and obtain GWF, which is then integrated into commercial contact lenses (Zhang et al. 2022).

GWFs also enable intraocular pressure (IOP) monitoring, as IOP changes cause eye expansion, stretching the GWF and altering its electrical resistance (Kim et al. 2019). A constant voltage applied across the GWF results in current changes corresponding to IOP variations. Experiments with porcine eyes, using saline injections to simulate IOP fluctuations, and three tonometers paired with GWF lenses showed a significant correlation between IOP changes and GWF resistance/current (Liu et al. 2021). The device demonstrated an average resolution of 6.8% per mmHg and a maximum of 7.3% per mmHg within a 0-10 mmHg IOP range. Although earlier research had a resolution of 0.6% per mmHg (ref. 24), this sensitivity is sufficient for practical use. The lens material also showed minimal strain, underscoring the value of biocompatible materials in IOP monitoring (Li et al. 2015).

### Poly(sulfobetaine methacrylate) (or PSBMA/SBMA)-based nanogel

Wang et al. developed a zwitterionic nanogel made of poly(sulfobetaine methacrylate) (PSBMA) for integration into contact lenses aimed at continuous eye care (Wang et al. 2021). PSBMA’s zwitterionic properties, including high hydrophilicity and antifouling characteristics, help improve comfort, reduce bacterial adhesion, and support long-term compatibility with the eye (Zhang et al. 2013; Gil et al. 2017). The material’s ability to bind water via electrostatic interactions enhances moisture retention and reduces protein adsorption, addressing common issues like dryness and irritation in prolonged contact lens use.

The nanogels were synthesized via reflux-precipitation polymerization in acetonitrile, yielding stable, uniform particles. Levofloxacin, an effective broad-spectrum antibacterial agent, was encapsulated to enable sustained drug release. The drug-loaded nanogels were then incorporated into contact lenses with varying drug concentrations to assess the relationship between drug loading and release (Vaisocherová et al. 2009; Kaveti et al. 2024; Karimi et al. 2024).

Three primary parameters-flexibility, cell viability, and water retention-were tested to ensure biocompatibility and mechanical suitability. The PSBMA nanogel lenses showed substantial flexibility, withstanding 150% strain without structural damage, indicating their comfort and durability (Vaisocherová et al. 2009; Kaveti et al. 2024). Cytotoxicity tests with mouse embryo fibroblast cells revealed no significant differences in viability between the nanogel lenses and control, confirming biocompatibility (Bai et al. 2014; Vaisocherová et al. 2009).

Water retention, assessed by equilibrium water content (EWC), demonstrated the lenses’ ability to maintain moisture, reducing dryness during prolonged wear (Dai et al. 2021; Kaveti et al. 2024). Additionally, drug release profiles showed sustained Levofloxacin release, offering consistent antibacterial action compared to the frequent administration required by traditional eye drops (Chen et al. 2016; Ma et al. 2021).

This PSBMA nanogel system could be adapted for broader ophthalmic applications, such as delivering intraocular pressure-lowering medications for glaucoma, providing more consistent therapeutic effects (Ma et al. 2021). Overall, PSBMA nanogel contact lenses offer a promising platform for sustained ocular drug delivery.

## Myriad shapes for smart contact lenses

Another important consideration in the fabrication of smart lenses for glaucoma treatment is the manner in which biocompatible materials are synthesized, particularly with respect to their shape (Liu et al. 2024). The structural configuration of these materials plays a crucial role in ensuring both their functionality and compatibility with the delicate structures of the eye (Seo et al. 2023). Additionally, the shape of the materials can influence the performance of the lenses, such as their ability to deliver medication, monitor intraocular pressure, or adapt to the eye’s natural curvature, which are all essential features for effective glaucoma management (Kim et al. 2020).

### Ring

**Fig. 4:**
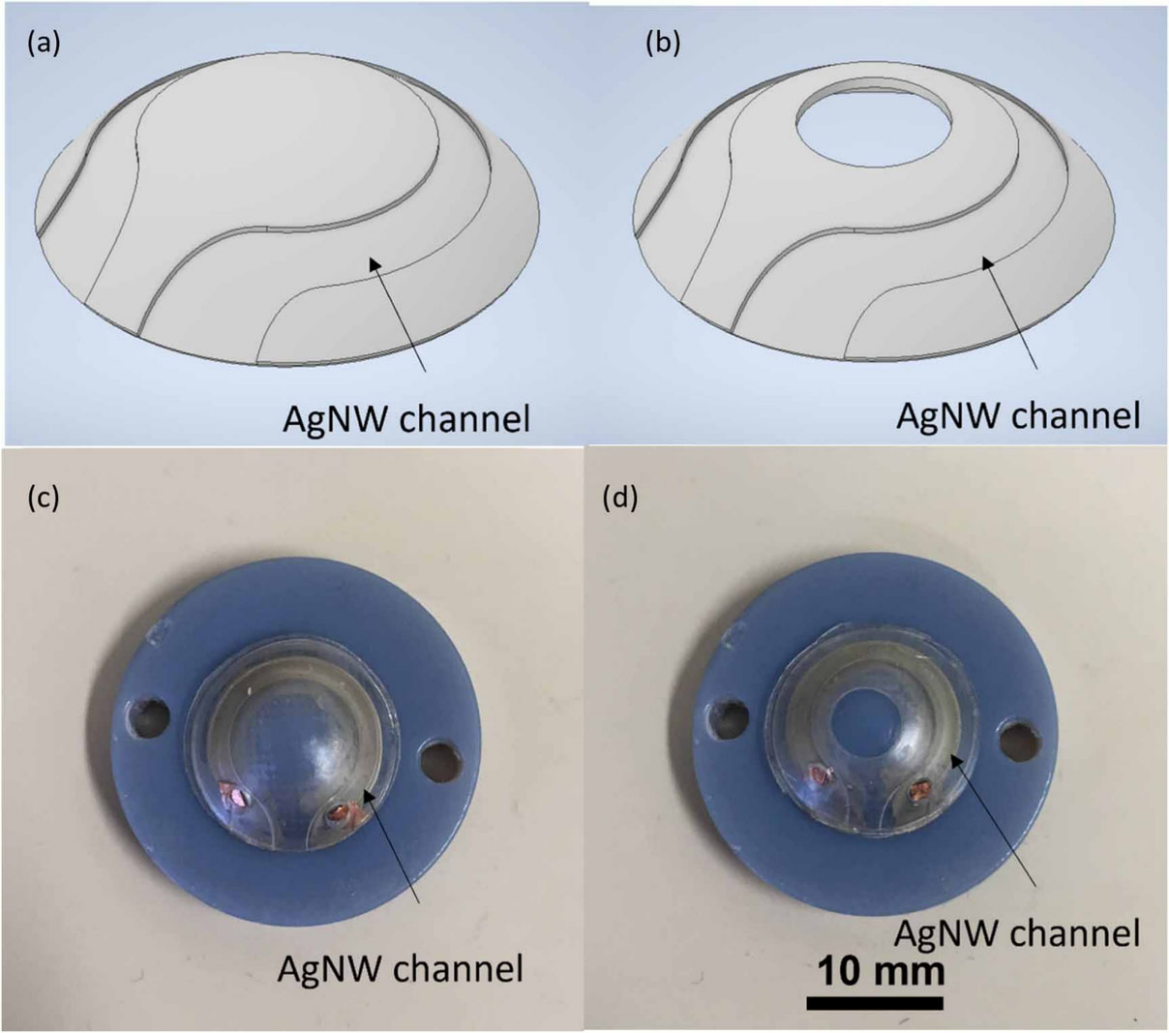
(a) (Top left) CAD design of a lens without a hole. (b) (Top right) CAD design of a lens featuring a 4.7 mm hole. (c) (Bottom left) Physical lens without a hole. (d) (Bottom right) Physical lens with a 4.7 mm hole. The blue plastic serves as a convex base for lens positioning and is not part of the sensor (Helgason and Lai 2022)

Helgason and Lai (2022) proposed a novel ring-shaped contact lens design demonstrated in Fig. 4 to improve intraocular pressure (IOP) sensing in smart lenses for glaucoma management. Traditional lenses with piezoresistive strain gauges often have limited sensitivity due to conventional designs.

The ring-shaped lens, featuring a central hole (2.7 mm or 4.7 mm diameter), amplifies circumferential strain as IOP changes, enhancing the sensor’s piezoresistive response by allowing eye expansion within the hole’s inner edge (Figure 4(b and d)).

Finite element analysis and experiments on porcine and PDMS model eyes confirmed increased sensitivity: the 2.7 mm hole improved sensitivity by 7.1%, and the 4.7 mm hole by 17.9%. This design also improves oxygen flow and minimizes visual obstruction.

The study highlights the potential of structural modifications for optimizing strain-gauge-based sensors in smart lenses for real-time IOP monitoring, suggesting applications for other ocular wearable devices (Helgason and Lai 2022).

### Moiré

Lee et al. (2020) developed a multifunctional contact lens for continuous intraocular pressure (IOP) monitoring and temperature-triggered drug release, offering an advanced solution for glaucoma management. This innovation addresses precise IOP monitoring and on-demand medication delivery.

**Fig. 5:**
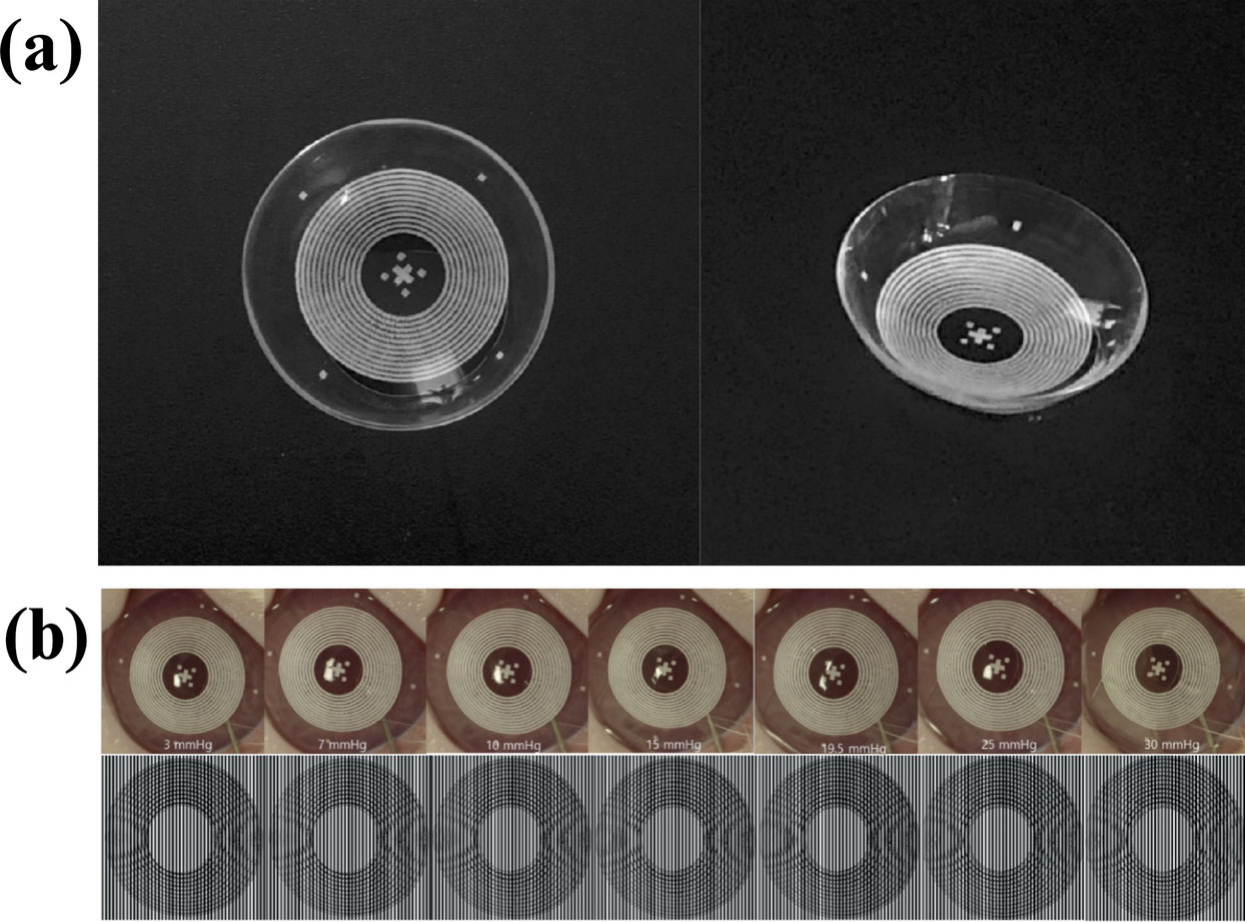
(a) A contact lens with an imprinted manufactured moiré pattern. (b) Intraocular pressure (IOP) monitoring using moiré pattern-based techniques in rabbits with glaucoma induced through continuous BSS (Balanced Salt Solution) injection. In the first row, contact lenses embedded with moiré patterns show IOP changes. In the second row, overlaid moiré patterns alongside virtual reference images implicitly reflect IOP variations (Lee et al. 2020). Se-Hee Lee, Kyung-Sik Shin, Jae-Woo Kim, Ji-Yoon Kang, and Jong-Ki Kim, Translational Vision Science & Technology, Vol. 9, Article 1, 2020; licensed under a Creative Commons Attribution-NonCommercial-NoDerivatives 4.0 International License

The lens uses a concentric moiré pattern that forms interference patterns in response to IOP changes (Figure 5(a)). Unlike electromechanical sensors, this optical method eliminates complex electronics, capturing eye surface deformation through a lens imprinted with moiré patterns. When overlaid with virtual reference images, these patterns provide real-time IOP data, detecting changes in the clinically significant range of 3-40 mm Hg. Additionally, the lens incorporates thermosensitive nanogels loaded with timolol, which release the drug at body temperature, triggered by IOP spikes. This ensures efficient, timely drug delivery without traditional eye drop schedules.

According to the rabbit glaucoma models tested by the researchers (Figure 5(b)), the moiré patterns demonstrated strong linearity with IOP ($R^2$ > 0.92). Drug release achieved sustained timolol delivery over seven days, with a cumulative dose of 10.6 $\mu$g/mL in aqueous humor, resulting in a 33% IOP reduction within hours. These results highlight the lens’s potential to integrate monitoring and treatment in a single device.

This study illustrates how optical sensing and temperature-responsive drug delivery can transform smart contact lenses into versatile therapeutic tools. For patients, this technology could offer consistent IOP control, fewer interventions, and reduced side effects. Future advancements may focus on scaling for human use, enhancing comfort and durability, and incorporating wireless data transmission for remote IOP monitoring. This research exemplifies how smart lenses can bridge diagnostic monitoring and personalized treatment, paving the way for autonomous ocular health management in chronic conditions like glaucoma (Lee et al. 2020).

### Serpentine

Kim et al. advanced ophthalmic diagnostic technologies by developing a stretchable corneal sensor for non-invasive, continuous glaucoma and intraocular pressure (IOP) monitoring. Unlike the ERG-Jet, which uses electrodes directly contacting the eye to measure retinal electrical activity, causing discomfort and limiting long-term use (Kim et al. 2021; Brown 1968), their design integrates a serpentine-patterned electrode into a contact lens.

**Fig. 6:**
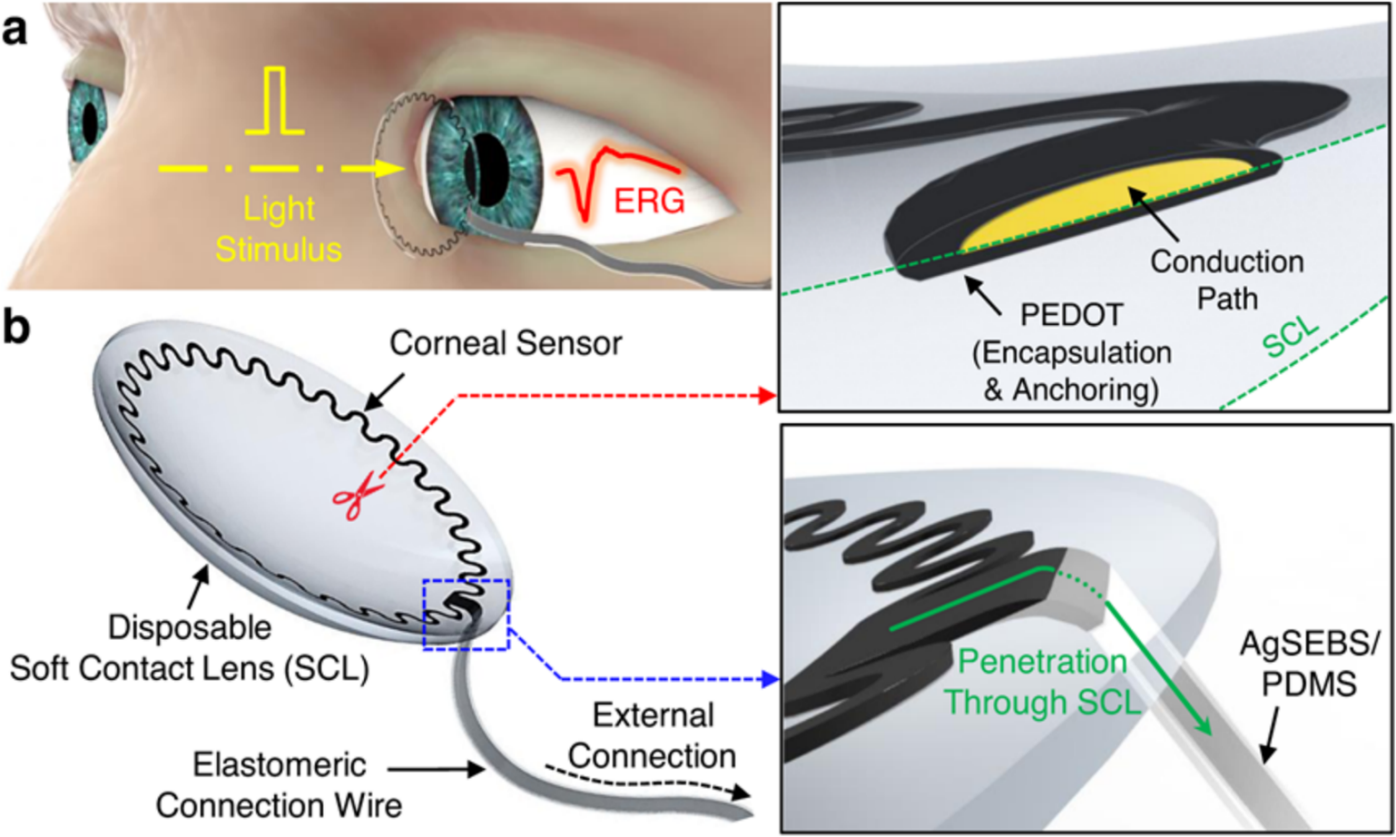
(a) Schematic representation of ERG recordings in response to light stimuli from a human eye using a corneal sensor. (b) Schematic representations of the corneal sensor, with inset images detailing the embedded encapsulation and anchoring layers (top panel) and its seamless integration with the connection wire (bottom panel) (Kim et al. 2021)

The electrode, located at the lens’s periphery, leaves the central region clear for light transmission (Figure 6(a-b)). Fabricated with conductive PEDOT doped with tosylate (Sekine et al. 2010; Asplund et al. 2009; He et al. 2019), it is encapsulated to enhance anchoring. Elastomeric wires composed of AgSEBS and PDMS seamlessly integrate with the lens, maintaining structural integrity. The lens remains functional under strain and typical wear conditions (Seo et al. 2023; Kim et al. 2021).

Mechanical analysis shows that under flipping, folding, stretching (40%), and expanding (10%), the sensor’s maximum strain ($\varepsilon _{max}$) remains below 10%, with deformation primarily occurring in the lens, preserving its mechanical properties (Kim et al. 2021). Integrated PDMS-based wires recorded retinal activity accurately, demonstrating performance comparable to the ERG-Jet (Kim et al. 2021).

This innovation highlights the importance of biocompatibility in smart contact lenses, enabling continuous monitoring of glaucoma with enhanced patient comfort and improving early detection and management of ocular diseases.

## Molecule sensing via smart contact lenses

Integrating molecular sensors into contact lenses offers significant potential for continuous intraocular pressure (IOP) monitoring in glaucoma care (Leonardi et al. 2003; Zhou et al. 2024). By detecting abnormal tear molecule concentrations, these technologies enable earlier glaucoma detection and precise IOP management, helping preserve vision (Shetty et al. 2024; Rajan et al. 2024; Sanchez and Martin 2019; Zhao et al. 2022; Bamgboje et al. 2021). Advances in tear sensor technology, already applied to glucose and sodium monitoring for conditions like diabetes and dry eye, provide a strong basis for glaucoma applications (Farandos et al. 2015; Shi et al. 2021; Lan et al. 2019). This innovation could transform glaucoma diagnosis and management, marking a pivotal step in preventive ophthalmic health.

### Sodium-sensitive contact lens

Badugu et al. (2021) developed a sodium-sensitive contact lens to diagnose ocular pathologies like dry eye disease (DED). Conventional DED diagnostic methods, such as the Schirmer test and total tear osmolarity measurements, offer limited insights as they focus on tear production or overall electrolyte concentration without isolating specific ions. This study addressed this limitation by creating a lens that selectively detects sodium ions, providing valuable diagnostic data.

To achieve this, the team synthesized three modified fluorophores-SG-C16, SG-LPE, and SG-PL-from Sodium Green. These fluorophores were tailored to bind to hydrophobic regions of silicone hydrogel (SiHG) contact lenses and exhibit sodium-dependent spectral properties. When integrated into the lenses, they produce fluorescence that varies in intensity and lifetime with sodium ion concentrations, enabling real-time, reversible sodium sensing (Badugu et al. 2021). Experimental results showed stable and specific fluorescence responses unaffected by tear proteins, with the fluorophores retaining sensitivity and remaining intact after repeated use, demonstrating their reliability in ophthalmology.

This innovation represents a significant advancement in wearable diagnostics, potentially revolutionizing tear fluid analysis. Future research could focus on fluorophores for additional ions, enhancing polymer compatibility, and conducting clinical trials. Multi-ion-sensitive lenses and portable readers may further support at-home, patient-driven diagnostics, paving the way for personalized eye care.

### MMP-9 sensing and intraocular pressure monitoring for glaucoma management

**Fig. 7:**
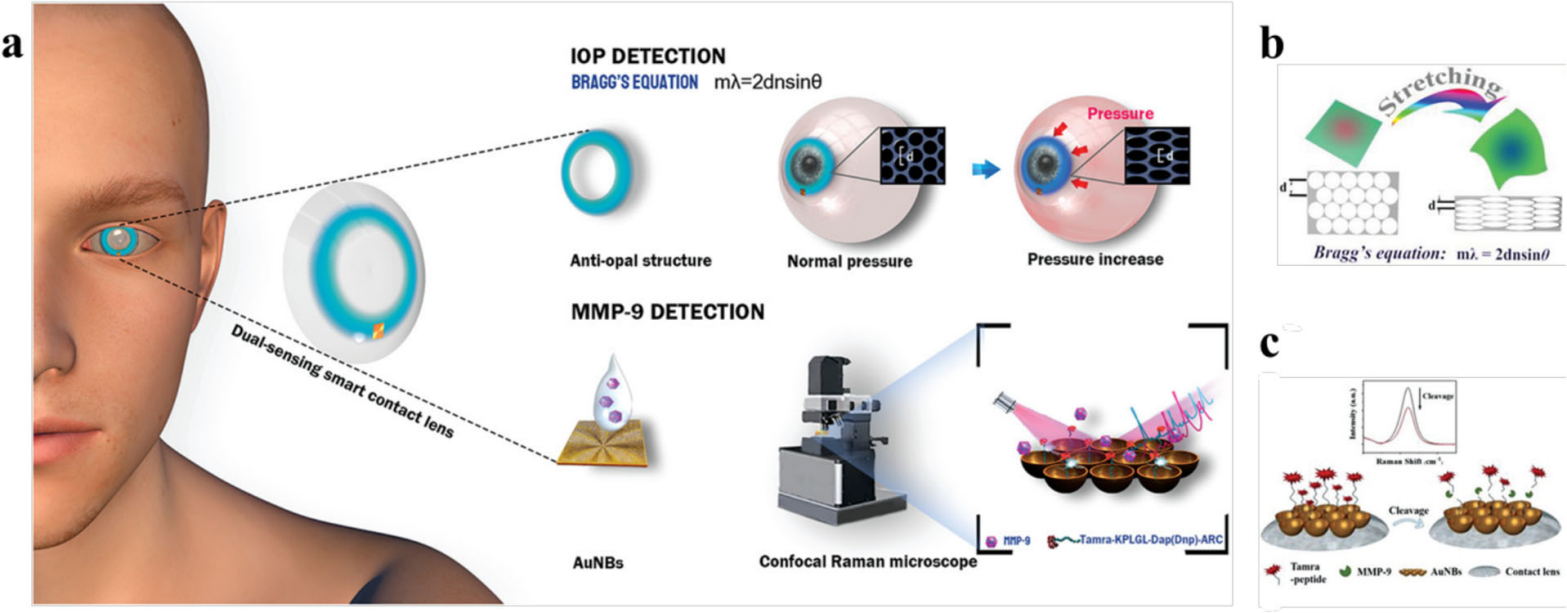
(a) Diagram of the dual-functional contact lens sensor, featuring an antiopal structure designed for intraocular pressure (IOP) monitoring and a peptide-functionalized gold nanobowl (AuNB) SERS substrate tailored for MMP-9 biomarker detection. (b) Schematic representation of the IOP monitoring mechanism using structural color contact lenses. (c) Schematic representation of the Tamra-pep cleavage by MMP-9 on the AuNBs SERS substrate attached to contact lenses. Ye et al. (2022)

Recent advancements in molecular sensing have introduced innovative methods for detecting matrix metalloproteinase-9 (MMP-9) in tears, a crucial biomarker for glaucoma diagnosis and monitoring (Shetty et al. 2024). While conventional methods rely on immunoassays, Ye et al. proposed a contact lens system combining MMP-9 detection with intraocular pressure (IOP) monitoring (Figure 7(a)), using biocompatible materials and a dual-sensing mechanism (Ye et al. 2022).

MMP-9 detection utilizes peptides functionalized within a gold nanobowl structure embedded on the lens surface, achieving a detection limit of 0.90 ng/mL. Peptide cleavage upon MMP-9 binding triggers a surface-enhanced Raman scattering (SERS) response, enabling precise quantification (Ye et al. 2022). The IOP monitoring system incorporates a ring structure that stretches with ocular pressure changes, causing a measurable color shift (Figure 7(b)). This shift aligns with Bragg’s equation ($\lambda = \frac{2dn\sin \theta }{m}$), where *d* is lattice spacing, *n* is the hydrogel refractive index, $\theta$ is the incident light angle, and *m* is the diffraction order (Ye et al. 2022; Liu et al. 2024).

Figure 7(c) demonstrates that Raman intensity measurements targeting MMP-9 show high specificity with minimal interference from other compounds, highlighting the sensor’s molecular precision. This dual-functional lens enables both biomarker detection and continuous IOP monitoring for glaucoma care. To ensure safe, long-term use, the lens was designed for biocompatibility, focusing on oxygen permeability and wettability (Ye et al. 2022; Xiang et al. 2023).

**Fig. 8:**
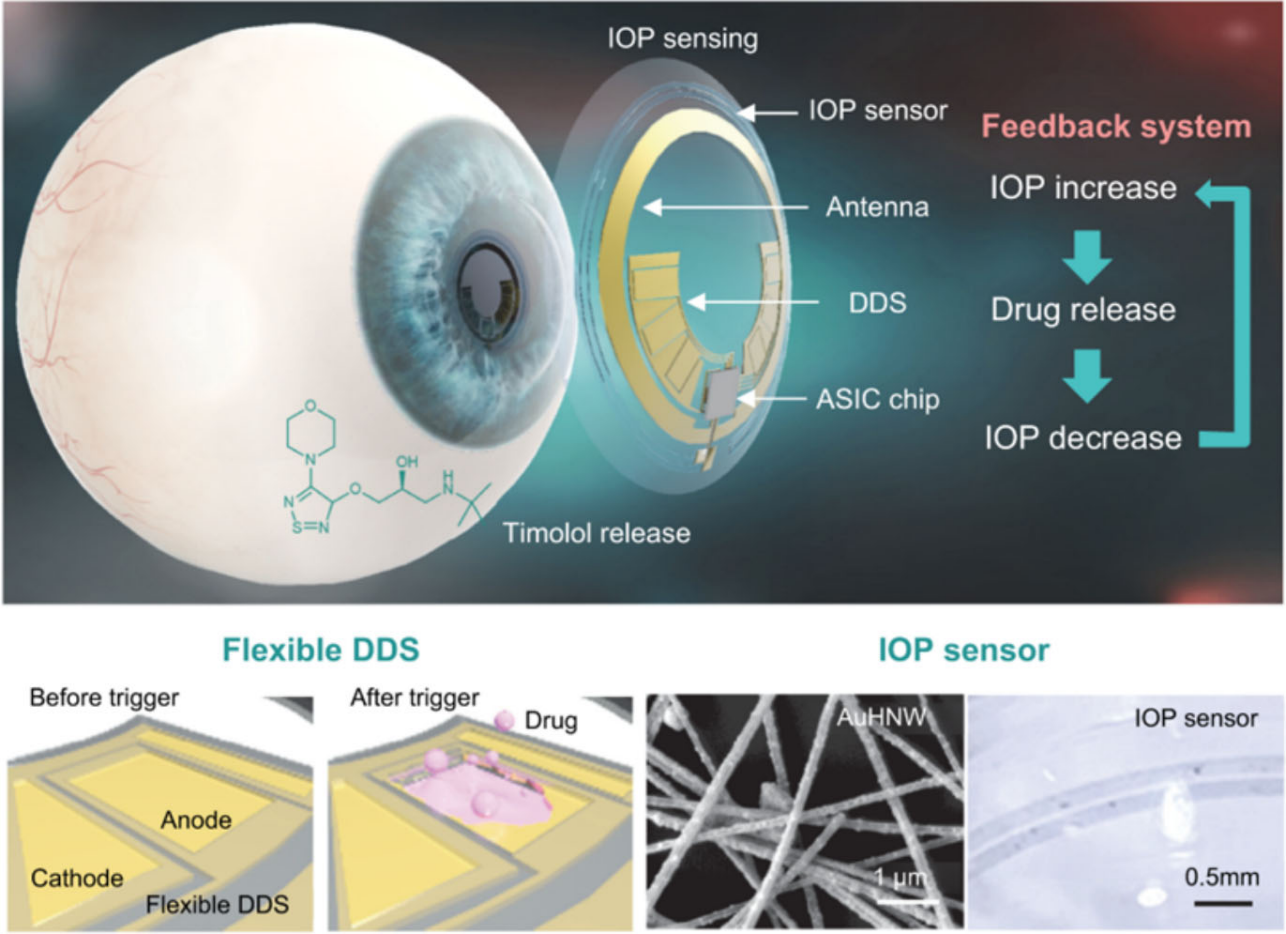
Schematic of the theranostic smart contact lens, which integrates an AuHNW-based IOP sensor, drug delivery system (DDS), and wireless circuitry for glaucoma treatment with real-time IOP sensing and timolol release (Kim et al. 2022)

### Continuous intraocular pressure (IOP) monitoring

Advances in sensor technology now enable continuous intraocular pressure (IOP) monitoring for glaucoma diagnosis. Kim et al. integrated an IOP sensor into a contact lens, enabling real-time IOP tracking through resistance variations caused by ocular deformation (Figure 8) (Kim et al. 2022). Moreover, an embedded application-specific integrated circuit (ASIC) converts resistance changes into IOP values.

Biocompatibility is essential for long-term use. Kim et al. evaluated this through cell culture studies on D-sorbitol poly(3,4-ethylenedioxythiophene) (PEDOT) and gold hollow nanowires, observing minimal cell death and comparable growth rates via fluorescent staining (Kim et al. 2022). Furthermore, optical microscopy further confirmed no significant corneal thickness changes after prolonged wear on rabbits. Sensor accuracy was validated against a commercial tonometer, showing a linear correlation between methods across test cases, demonstrating reliability (Maeng et al. 2020; Kim et al. 2021).

These smart contact lenses provide continuous, reliable IOP monitoring with minimal biological impact, advancing wearable technology for glaucoma management. Despite these innovations, the rigidity of sensor-embedded lenses limits long-term wearability. Flexible circuit designs, discussed in the next section, address this by reducing stiffness, enabling 24-hour monitoring, and enhancing patient comfort. Ongoing research aims to optimize functionality and usability further.

## Soft smart contact lenses with integrated electronic elements

Smart soft contact lenses (SSCL) are an innovative technology for ocular health, designed to continuously monitor intraocular pressure (IOP), a key factor in glaucoma management (Quintero et al. 2017; Park et al. 2018; Kouhani et al. 2020; Kim et al. 2021). By integrating micro-sensors and wireless systems, SSCLs enable non-invasive, real-time IOP tracking, overcoming the limitations of traditional periodic measurements (Shean et al. 2024; Leonardi et al. 2004; Ma et al. 2021). This advancement, driven by materials science and miniaturized electronics, has the potential to transform glaucoma care with early detection and improved treatment strategies (Seo et al. 2023; Abdulamier et al. 2024).

### Sensimed triggerfish contact lens

The Sensimed Triggerfish lens, a silicone soft contact lens with strain gauges, a microprocessor, and an antenna, was among the first to enable continuous 24-hour intraocular pressure (IOP) monitoring, addressing a key need in glaucoma care (Vitish-Sharma et al. 2018; Beltran-Agulló et al. 2017; Xu et al. 2016). Dunbar et al. reviewed its safety and efficacy (Dunbar et al. 2017). An adhesive antenna powers the lens and transmits data to a portable recorder, capturing 86,400 readings daily for detailed analysis via Bluetooth. While effective for short-term monitoring, it has mild side effects like inflammation and blurred vision, limiting long-term use. Despite these drawbacks, the Triggerfish lens laid the groundwork for advanced smart soft contact lenses (SSCLs).

### Smart soft contact lenses for reliable intraocular pressure monitoring

**Fig. 9:**
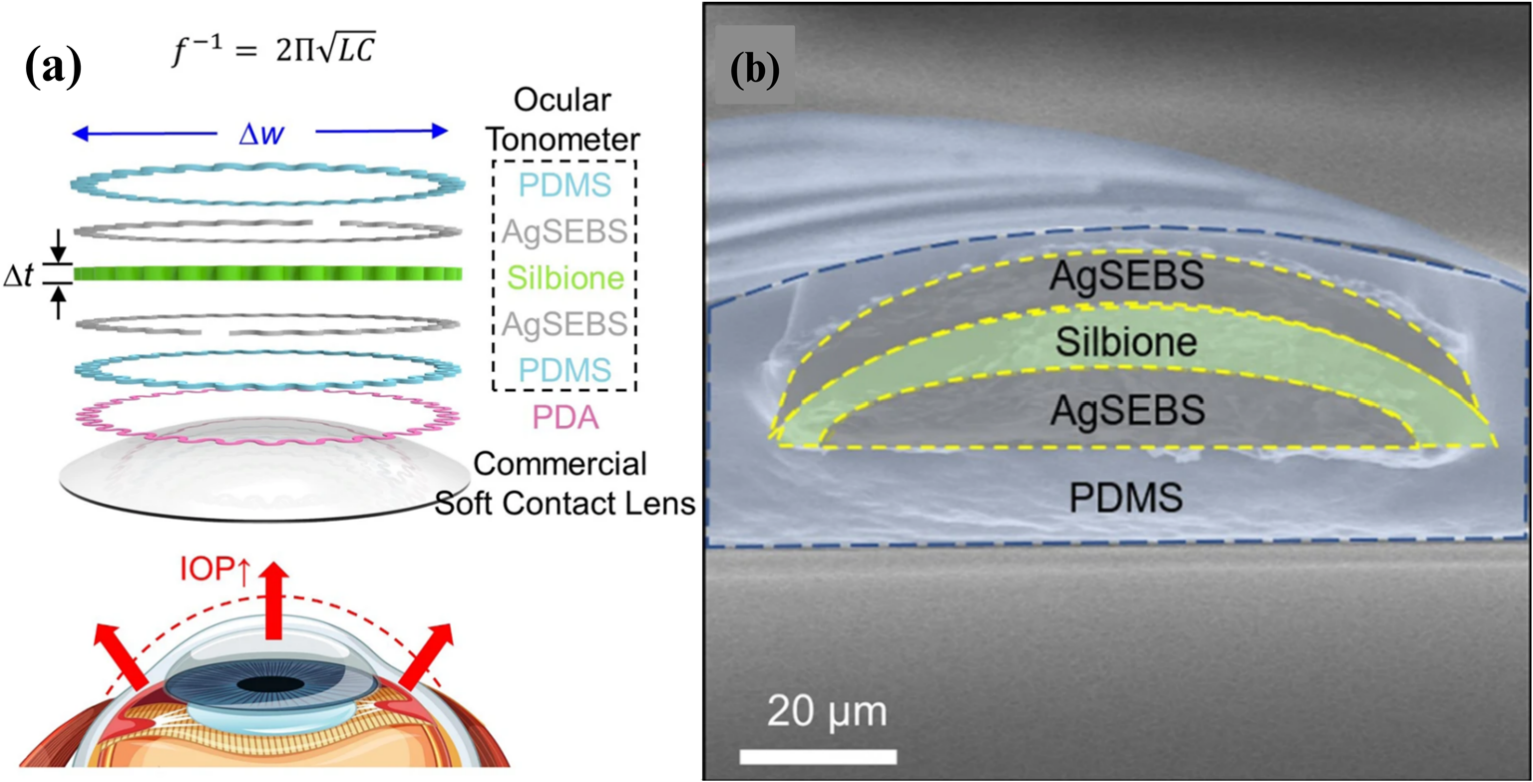
(a) The schematic of the different layers of the proposed SSCL. (b) the SEM image of the SSCL (Zhang et al. 2022)

Zhang et al. developed a smart soft contact lens (SSCL) with an integrated intraocular tonometer (Zhang et al. 2022). The device features a multi-layer structure for functionality and biocompatibility. The outer PDMS layers enhance biocompatibility, while SEBS layers embedded with silver flakes serve as conductive traces. A central liquid silicone rubber layer provides electrical insulation. These components form an RLC series resonant circuit, the core of the tonometer, as shown in Fig. 9(a, b).

The ocular tonometer is securely bonded to the contact lens using a polydopamine adhesive, leveraging its polymerization for durability. The SSCL monitors intraocular pressure (IOP) by detecting changes in the eye’s curvature radius. Increased IOP stretches the lens, altering the RLC circuit’s inductance and capacitance, which decreases the resonance frequency, described by:

$$ f^{-1} = 2\pi \sqrt{LC}. $$

Tests on three eyes showed a consistent linear relationship between resonance frequency and IOP, minimizing variability and ensuring reliability. The lens demonstrated excellent biocompatibility, maintaining functionality under conditions such as stretching, disinfecting, dehydration, heating, and 30-day saline soaking. Resonance frequency variation remained minimal, ensuring robust measurements. Additionally, the adhesive exhibited strong durability, making it suitable for long-term use. IOP readings were consistent with commercial tonometers, validating the lens’s practical application.

### Wireless theranostic contact lens (WTCL)

Wireless theranostic contact lenses (WTCL) represent a breakthrough in glaucoma care, combining intraocular pressure (IOP) monitoring and drug delivery in one device. Yang et al. developed a smart lens that continuously measures IOP while administering therapeutic agents, offering a dual-function solution for glaucoma management (Yang et al. 2022).

**Fig. 10:**
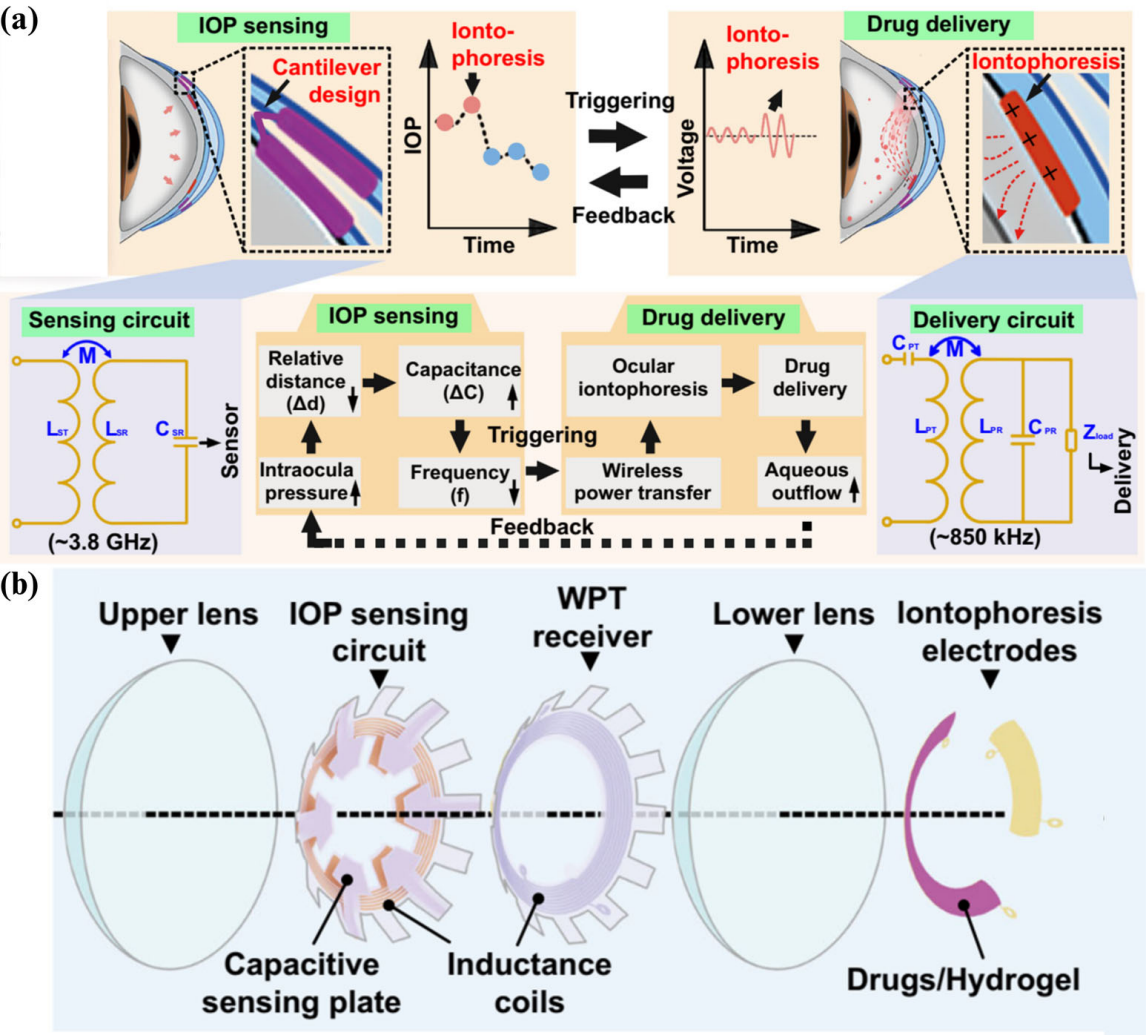
(a) Schematic illustrating the wireless operation for IOP monitoring and on-demand drug delivery in a minimally invasive manner. The soft device, designed as a double-layer contact lens, incorporates an LCR circuit and a wireless power transfer (WPT) receiver. These components are wirelessly linked to an external integrated antenna, enabling IOP signal recording and activation of iontophoresis for drug release when required. The inset figures highlight key components of the IOP sensing and drug delivery units. (b) Exploded view of the WTCL structure (Yang et al. 2022)

**Fig. 11:**
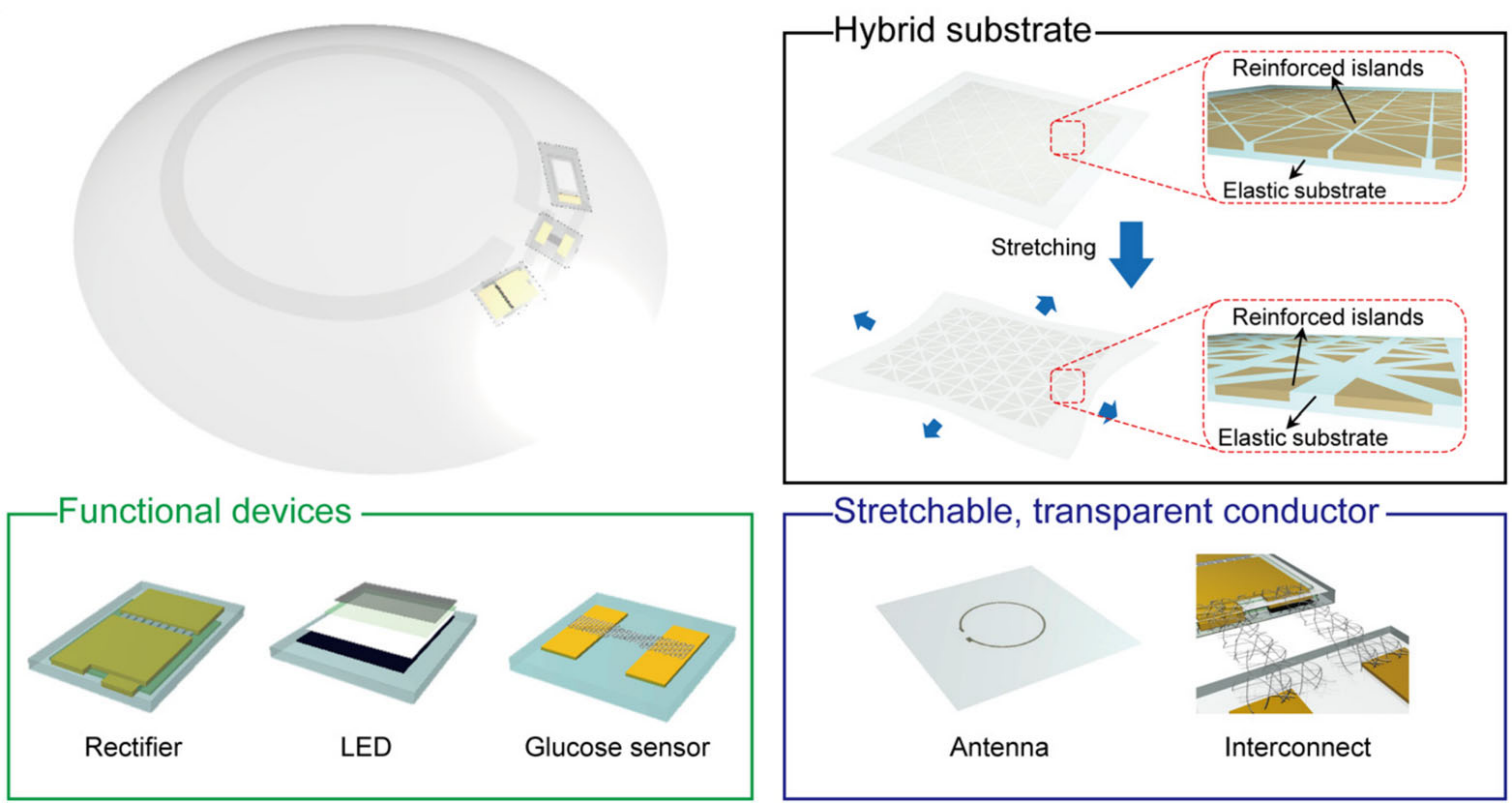
Schematic representation of the soft, smart contact lens featuring a hybrid substrate, integrated functional devices (rectifier, LED, glucose sensor), and a transparent, stretchable conductor for the antenna and interconnects (Park et al. 2018)

The WTCL integrates an embedded RLC circuit for wireless IOP monitoring. Its sensing system, illustrated in Fig. 10(a), features a snowflake-shaped design with six capacitive sensing plates, five inductance coils, and six reference plates in the upper lens. These plates form a variable capacitor with the lower lens, separated by an air dielectric layer. Increased IOP compresses this layer, raising capacitance and lowering the RLC circuit’s resonance frequency, which is wirelessly detected by an external coil (Yang et al. 2022).

For drug delivery, iontophoresis electrodes beneath the lower lens release medications like brimonidine from a hydrogel layer into the cornea. This wireless system ensures sustained, controlled drug delivery to lower IOP. Combining real-time monitoring with targeted therapy, WTCLs promise a transformative, non-invasive approach to glaucoma care, with studies affirming their potential for clinical ophthalmology applications.

### Soft, smart contact lenses with integrations of wireless circuits, glucose sensors, and displays

Park et al. (2018) developed a smart contact lens with a glucose sensor integrated into an RLC circuit to monitor glucose levels in tears for diabetes management (Park et al. 2018). The system, shown in Fig. 11, includes a rectifier circuit, an LED indicator, a glucose sensor, and a wireless power antenna. When tear glucose exceeds a set threshold, the LED provides a warning, enabling timely intervention. The antenna wirelessly receives alternating current (AC) signals, converted to direct current (DC) by the rectifier to power the sensor and LED.

The glucose sensor detects glucose levels by measuring resistance changes; higher glucose concentrations decrease resistance, allowing more current to flow and power the LED. To address mechanical strain challenges in integrating electronics into the soft lens structure, the authors designed a hybrid substrate. This substrate, made from a photocurable optical polymer, featured mechanically reinforced islands patterned onto a copper layer via photolithography. After applying a silicone elastomer coating, the copper layer was removed, creating a flexible substrate to support the lens’s electronic components.

This innovation highlights the fusion of biomedical engineering and wearable technology for non-invasive glucose monitoring. The hybridization of soft and rigid elements in wearable devices offers promising advancements in managing chronic conditions like diabetes (Imani et al. 2016).

## Contact lenses as drug delivery systems

Nanotechnology, particularly nanogels, offers a promising advancement in ocular drug delivery for glaucoma management (Onugwu et al. 2023). These cross-linked polymer networks encapsulate therapeutic agents, enabling controlled release. When integrated into IOP-monitoring contact lenses, nanogels provide a synergistic mechanism for continuous glaucoma care by responding to IOP changes and releasing medication directly to the eye, enhancing efficacy and patient outcomes (Peng et al. 2012).

For example, a moiré-patterned lens integrated a porous nanogel matrix synthesized using HEMA, ethylene glycol dimethylacrylate, and 3-(trimethoxysilyl)propyl methacrylate with the surfactant Silmer A008-UP, polymerized under UV light (Lee et al. 2020). This lens delivered Timolol, demonstrating sustained drug release in rabbit models, significantly reducing IOP over time. By enhancing corneal drug absorption, this approach surpasses traditional eye drops.

Nanotechnology-infused contact lenses offer innovative solutions for glaucoma treatment by combining controlled drug release with IOP monitoring. Together with technologies like ocular inserts and micro-drainage devices, they hold potential for more effective and comprehensive glaucoma management (Sultana et al. 2011; Fardoost et al. 2024).

## Glaucoma treatment using contemporary technologies for the management of other ocular diseases

Recent innovations in ocular technologies, initially developed for other diseases, show potential for advancing glaucoma management through improved IOP monitoring and control. Bailey et al. (2021) introduced a smart contact lens system for presbyopia that uses an infrared-triggered liquid crystal lens to adjust focal power (Bailey et al. 2022). While designed for presbyopia, this MEMS-based approach could be adapted for glaucoma, combining vision enhancement with IOP stabilization. Similarly, Lee et al. explored near-infrared (NIR) LED contact lenses for diabetic retinopathy, which track retinal cellular activity (Lee et al. 2022). Adapted for glaucoma, NIR technology could monitor glaucomatous cell degeneration, aiding early detection and disease tracking. These cross-disciplinary innovations highlight shared technological advancements in ocular research, offering pathways to enhance continuous IOP monitoring and personalized glaucoma treatment.

## Conclusion

Smart contact lenses represent a transformative innovation in glaucoma care, tackling challenges in IOP monitoring and treatment. By combining advanced materials, biosensors, and wireless communication, these lenses provide continuous, real-time IOP data, improving diagnostic accuracy and enabling timely intervention. Integrated drug delivery systems address medication adherence issues through controlled, sustained therapeutic release, enhancing efficacy and slowing optic nerve damage.

Beyond glaucoma, these lenses’ ability to detect biochemical markers and manage other ocular conditions broadens their potential for personalized eye care. However, challenges like long-term biocompatibility, comfort, and affordability remain. Addressing these through improved designs, materials, and cost reductions is vital for widespread adoption.

By integrating real-time monitoring, personalized treatments, and improved compliance, smart contact lenses could revolutionize glaucoma management, significantly reducing vision loss and the global burden of blindness.

## Data Availability

No datasets were generated or analysed during the current study.
